# Reply to “On the origin of molecular oxygen in cometary comae”

**DOI:** 10.1038/s41467-018-04943-w

**Published:** 2018-07-03

**Authors:** Y. Yao, K. P. Giapis

**Affiliations:** Division of Chemistry and Chemical Engineering, California Institute of Technology, 1200 E. California Blvd, Pasadena, CA 91125 USA

Laboratory experiments suggest that the molecular oxygen, detected in the coma of comet 67P, is produced in part by abstraction reactions of cometary water ions at exposed surfaces on the nucleus and on the spacecraft. While production rates are likely small relative to water, O_2_ formation on the spacecraft, near the spectrometer used to detect O_2_, questions what its measured abundance means, and renders conclusions of a primordial origin premature.

Heritier, Altwegg, Berthelier (HAB) et al. discount the contribution of our proposed Eley-Rideal (ER) reaction mechanism^1^ to the observed O_2_ abundance^2^ in the 67P/G–C coma by positing that: (1) the flux of energetic water-group ions (H_2_O^+^, H_3_O^+^, and OH^+^) hitting the nucleus is not sufficient to produce the observed O_2_ signal, and (2) there are no instrumental effects in response to energetic O_2_^−^ ions and energetic O_2_ neutrals entering the DFMS.

The ion flux deficiency was conceded in our paper^1^. However, HAB et al. offer a new argument that shifts the debate. They show that, of the 2 water-group ion populations^3^ reaching Rosetta, the (50–300 eV) “accelerated” water ions originating in the extended coma exhibit a peak in flux perfectly out-of-phase with the H_2_O and O_2_ densities measured at the spacecraft between March 6 and 23, 2016. This anticorrelation is projected to also hold for any O_2_ produced when the accelerated H_2_O^+^ ions subsequently reach the nucleus. Assuming that ER reaction products are not trapped on the nucleus surface, the anticorrelation makes a compelling case against the accelerated water ions being the main driver for O_2_ production. The culprit flux must indeed be well-correlated with the neutral gas signal at ROSINA-COPS.

**Fig. 1:**
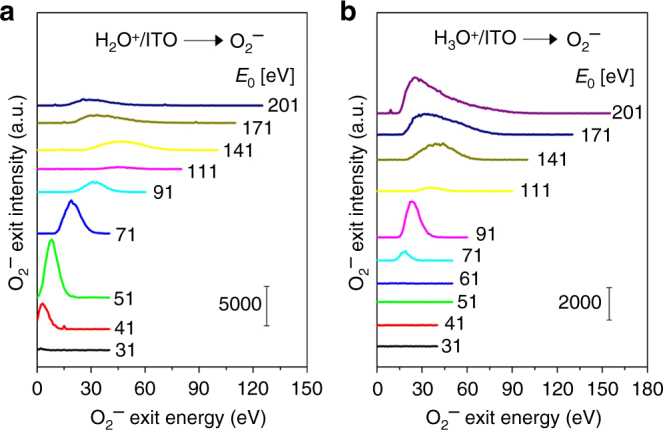
Production of O_2_^−^ from energetic H_2_O^+^ and H_3_O^+^ bombardment of ITO surfaces. Energy distributions of O_2_^−^ scattered from a thick layer of conductive indium–tin oxide following bombardment by **a** H_2_O^+^ and **b** H_3_O^+^ ion beams at various incidence energies (*E*_0_). Scattering geometry: 45° angle of incidence and 45° angle of exit. The ITO layer was deposited on a Cu sample by magnetron sputtering of a commercial high-purity ITO target

In contrast, the "cold” water ions are correlated with O_2_. Though not mentioned by HAB et al., the anticorrelation does not hold for the more abundant “cold” water-group ions^3^, produced in the space between the 67P nucleus and Rosetta, and arriving at the spacecraft with energies between 10 and 50 eV. Depending on heliocentric distance, these newly formed water ions experience the solar wind convective electric field^3,4^, or the ambipolar electric field^5^ of the inner cometary plasma and gain energy as they move away from the nucleus. Upon reaching Rosetta, the negative spacecraft potential accelerates them further to impinge on exposed spacecraft surfaces at energies that can be measured by the RPC-ICA^3^ and ion and electron sensor (IES)^4,6^ instruments. Unlike the sporadic arrival of accelerated ions, “the cold population is almost always present” at Rosetta^3^ during the entire mission, tracking well the averaged neutral gas density preperihelion and postperihelion^7^. With respect to flux oscillations, Goldstein et al.^6^ present timed cold-ion arrival data for September 10, 2014, demonstrating in Fig. 1 that the flux of these ions peaks contemporaneous with the neutral gas density measured by COPS. More intense IES signal is seen for 10–50 eV ions between October 17 and 21, 2014, when the ion and neutral gas peaks are perfectly synchronized, see Fig. 5 in Galand et al.^8^. Langmuir probe derived ion densities also exhibit peaks perfectly coincidental with the neutral gas density between 14 and 22 October 2014, see Fig. 1 in Edberg et al.^9^. Remarkably, the October 17–23, 2014 period coincides with the strongest linearity (*R* = 0.97) seen between O_2_ and H_2_O DFMS signals^2^. The correlation holds even closer to perihelion, see Fig. 2 in Volwerk et al.^10^ for June 7, 2015. Thus, it appears the cold water-group ions are well-correlated with the H_2_O and O_2_ neutral gas densities throughout the mission.

Rosetta emits its own O_2_. “Cold” water ions possess enough kinetic energy to also drive ER reactions on exposed spacecraft surfaces—the threshold for neutral O_2_ formation in H_2_O^+^ collisions with oxygen atoms on metal surfaces is estimated to be in the 5–8 eV range. These surfaces, include aluminium frame components, photovoltaic (PV) panels, and multi-layer insulation (MLI) protection. The PV panel windows are coated with transparent conductive indium–tin oxide (ITO), while the MLI has a top layer consisting also of conductive ITO (for uniform spacecraft potential) (M.G.G.T. Taylor & A.I. Eriksson, personal communication). Thus, a substantial surface area of ITO is exposed to and bombarded by water-group ions with energies between 10 and 50 eV. Figure 1 presents new results from scattering of energetic H_2_O^+^ and H_3_O^+^ on ITO surfaces under identical conditions to our original studies on cometary material analogues^1^. As in that case, we find that O_2_^−^ is produced readily on ITO, in fact with a lower H_2_O^+^ incidence energy threshold than that observed for scattering on SiO_*x*_ or FeO_*y*_ (Al-oxide behaves similarly). O_2_^+^ and neutral O_2_ are also co-produced (not shown) with varying kinetic energies and states of excitation. This experiment suggests that the “cold” water ions bombarding Rosetta produce O_2_ in situ, thus populating the gas cloud around the spacecraft with O_2_. Can any of this O_2_ be detected by the DFMS? This phenomenon is arguably equivalent to outgassing of the spacecraft, which has been shown^11–13^ to lead to detectable signal after many years of space travel, even when the DFMS is not in direct line-of-sight of the outgassing sources (e.g., during spacecraft maneuvers or other payload operations). Indeed, Beth et al.^13^ rationalized a false-positive detection of NH_4_^+^ by the DFMS on the grounds that “the gas cloud around the spacecraft may be contaminated by Rosetta itself.” Based on other background gas detection experiments, Schläppi et al.^11^ were the first to wonder whether “the spacecraft is surrounded by a significantly denser atmosphere that enhances the collision frequency and thus increases the return flux.” Given that the DFMS can detect spacecraft outgassing emissions far away from the comet, we see no reason why some of the O_2_ produced locally, while in orbit around the comet, will not make it into the DFMS.

Do ion-Rosetta collisions produce enough O_2_? The argument circles back to ion flux, albeit in H_2_O^+^ collisions with Rosetta surfaces. “Cold” water-group ion flux has been measured to be 2 orders of magnitude larger than that of “accelerated” water ions^3^ with the caveat that it may be underestimated “due to the limited field of view of the instrument”^7^. Though more significant, this flux is still too low (roughly by 100×) to justify the measured O_2_ abundance. However, O_2_ is now produced proximal to the DFMS, expanding the possibility of an instrumental effect. Can locally produced O_2_ entering the DFMS be ionized more efficiently than cometary O_2_? An important difference with O_2_ formed on Rosetta vs. the nucleus is the state of excitation of the molecule. ER reactions produce rovibrationally hot molecules, often also electronically excited (e.g., Rydberg states)^1^. Such excited O_2_ molecules (e.g., long-lived low lying singlet states) are more likely to survive the transit time into the DFMS ionizer when produced in its vicinity. Vibrationally and electronically excited O_2_ states have lower energy threshold and larger cross-section for electron impact ionization than the ground state^14^. Bottom line, excited O_2_ molecules entering an ionizer will produce more detectable O_2_^+^ ions than ground-state O_2_ neutrals.

Why does O_2_ appear to follow the *r*^*-*2^ Haser law? O_2_ yield in ER reactions depends on both flux and energy of the incident H_2_O^+^, where the ion energy is determined effectively by the spacecraft potential. While the “cold” water ion flux follows a 1/*r* scaling law (*r* = cometocentric distance)^3^, the O_2_ flux will exhibit a different *r* scaling because of the convoluted energy dependence. The spacecraft potential is determined by the balance between ions and electrons arriving at its surfaces, whose fluxes depend on cometocentric distance and latitude^15^. As a result, the spacecraft potential exhibits generally a decaying dependence on *r*, which transfers to the ion energy gained when traversing the sheath. The convoluted ion flux and energy dependencies on cometocentric distance produce a 1/*r*^*n*^ scaling, where *n* > 1. Thus, O_2_ signal may exhibit a scaling closer to the Haser law for entirely different reasons than those assumed by HAB et al.

Has the DFMS been calibrated for O_2_? None of the published papers^16–18^ and Ph.D. theses^19,20^ on DFMS operation and characterization contains any calibration data for O_2_, neither to energetic ions (O_2_^−^, O_2_^+^), nor to energetic O_2_ neutrals, nor to excited states of O_2_. Only background trace amounts of thermal O_2_ have been detected^19^. In his Ph.D. thesis, Schläppi^19^ presents calibration data to energetic Ne^+^ ions, but includes no such experiments with O_2_^+^ ions. An instrumental effect cannot be ruled out without knowledge of the DFMS response to energetic or excited O_2_.

In conclusion, laboratory scattering experiments of H_2_O^+^ on ITO surfaces suggest that ER reactions may produce O_2_ on Rosetta surfaces from “cold” water-group ions. Given the prevalence of the cold ion population, this phenomenon resembles intensified spacecraft outgassing. Therefore, some of the in situ produced O_2_ must contribute to the overall O_2_ signal detected. The magnitude of the contribution depends not only on the number density but also on the state of excitation of the O_2_ molecules entering the DFMS. Without instrument calibration, the actual level of cometary O_2_ cannot be established.
